# Supplementary material for: Continental-scale assessment of spatial food market accessibility in Africa using open geospatial data
Source: Sci Rep. 2026 Jul 4;16:30738. doi: 10.1038/s41598-026-59806-y (PMC13634922; doi:10.1038/s41598-026-59806-y)
Supplement: Supplementary file 1 [file 41598_2026_59806_MOESM1_ESM.pdf]

Continental-scale assessment of spatial food market accessibility in Africa using open geospatial data

1 Cumulative accessibility and entropy using walking travel times

Because walking is the default mode of mobility for many rural African households, we recomputed cumulative accessibility (number of markets reachable within 60min) and entropy (spatial distribution of reachable markets) using a walking-only friction surface. Results mirror those obtained for motorised travel, though accessibility values are markedly lower and inequality gradients steeper (see Fig. 1).

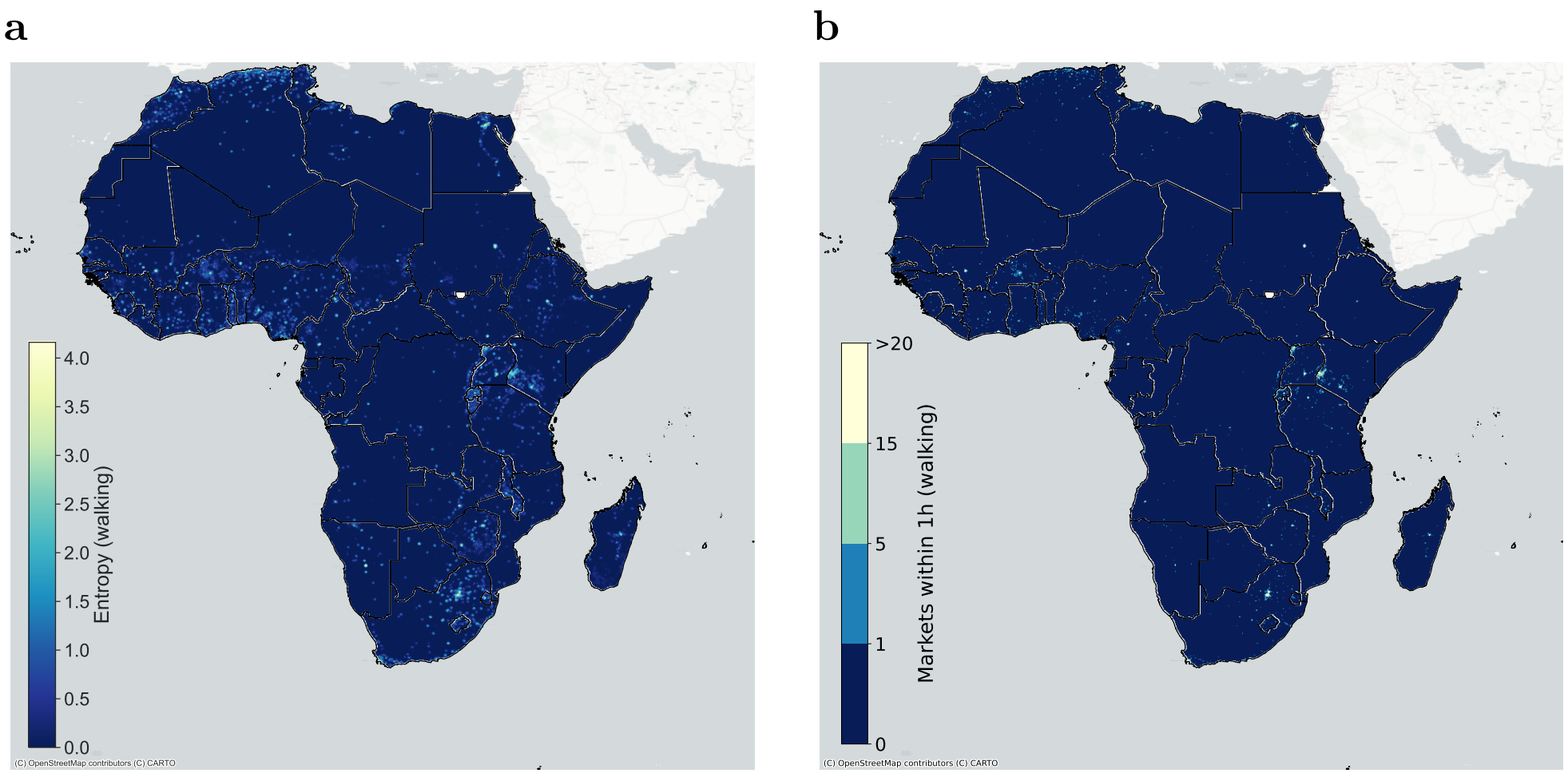

Fig. 1 Walking-only accessibility surfaces for entropy and number of markets. Maps for Africa showing **(a)** Entropy market distribution and **(b)** Number of markets within 1 hour, both computed using the walking-only friction surface. Both panels use the same 30-arc-second (≈1km) resolution as in the text.

2 Correlation between accessibility metrics and sensitivity analysis

To test the coherence of our three accessibility indicators, travel time to the closest market, cumulative accessibility, and entropy, we computed pair-wise Spearman rank correlations (population-weighted medians) at both country and pixel scales. In Fig. 2 panel **a** we observe the correlation matrix of the population-weighed medians of the accessibility metrics at the country level. In panel **b**, we increase the resolution of the correlation analysis at the pixel level. In this case, firstly, we compute the rank correlation coefficient for every country. Secondly, we calculate the average of the correlation coefficients across all countries in Africa as well as the average of the p-values. Results still display significant correlations, although, expectedly, the magnitude of the correlation decreases significantly due to the increased resolution.

In Fig. 2, panels **c** and **d** show the sensitivity analysis of the number of markets within a given travel time (cumulative accessibility), changing the threshold radius for Ethiopia. More specifically, in **c** we display the population average with the standard deviation and the maximum value, and in **d** we portray the relative variation (similar to a derivative, see eq. 1) of the population average. We chose to study the sensitivity of this metric in Ethiopia because it is a country with enough data, both from OSM and the WFP, to perform an appropriate analysis.

$$y'_i = \frac{y(x_{i+1}) - y(x_i)}{x_{i+1} - x_i} \tag{1}$$

In eq. 1, x is the travel time threshold, y is the population average and i is the index of the time threshold sequence.

The results in Fig. 2 **c** and **d** support the robustness of the proposed 30-minute motorized travel time threshold for the cumulative accessibility. Varying the travel-time threshold from 5 to 180 min in Ethiopia, mean accessible-market count remains stable around x=30min, and the derivative of the mean approaches zero in the 25–40min window, validating our chosen threshold.

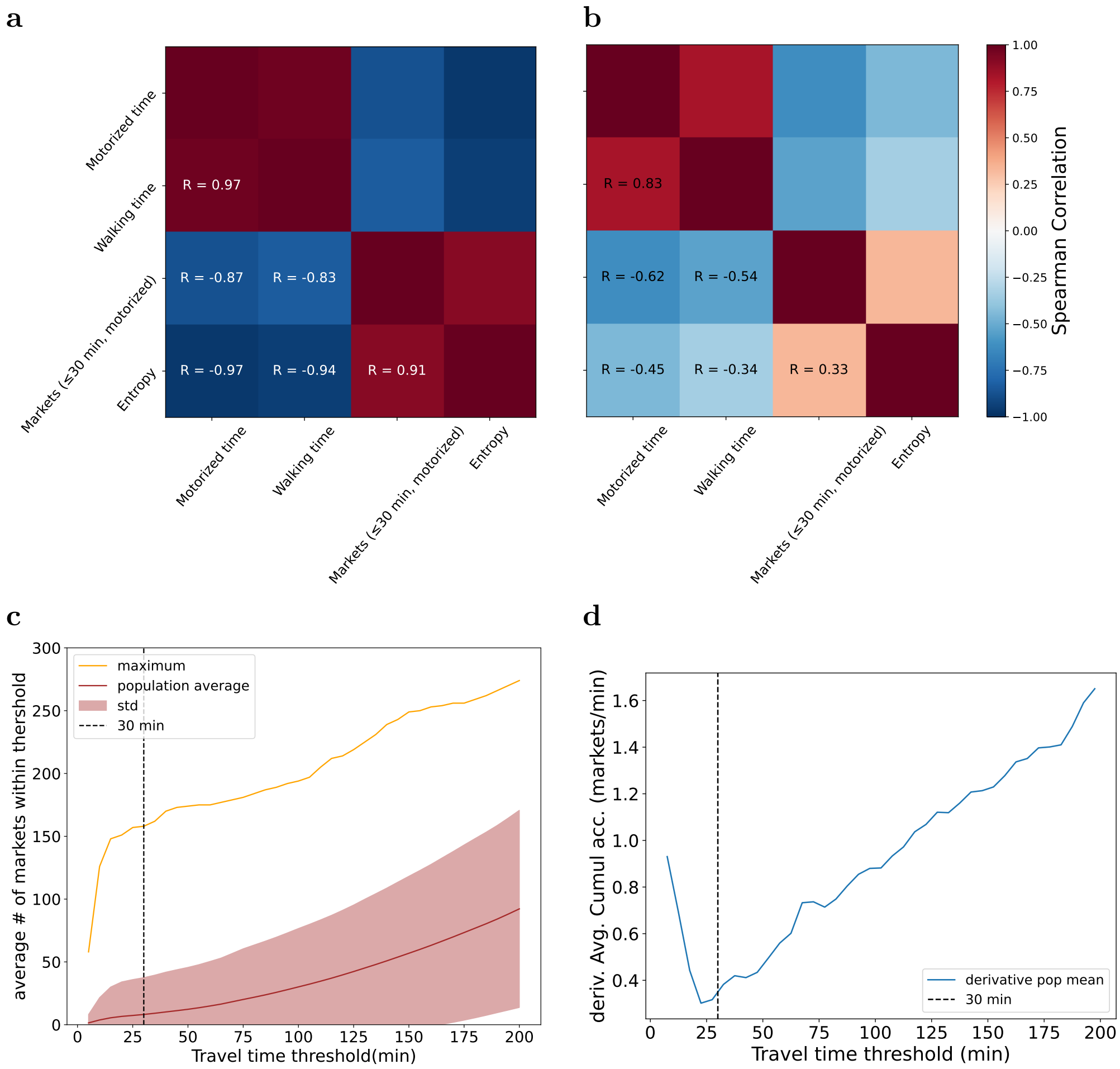

Fig. 2 **(a)** Matrix displaying the Spearman correlation between population-weighted median accessibility values at the country level. **(b)** Matrix displaying the Spearman correlation at the pixel level. For each country, we computed the rank correlation coefficient. The value displayed is the average of such coefficients across all African countries. **(c)** Evolution of different statistics of the cumulative accessibility with the threshold travel time value in Ethiopia. **(d)** "Derivative" of the average and population average of the cumulative accessibility with respect to the travel time threshold in Ethiopia.

3 Population divided in rural-urban classes within UN Africa Subregion

Table 1 disaggregates the population obtained by Worldpop in 2020 by urbanisation class (Urban, Suburban, Rural, Hinterland) for each of the five U.N. African sub-regions. Percentages highlight how Western and Eastern Africa are dominated by suburban settlements, whereas Middle Africa retains the largest hinterland share.

Table 1 Population in urban-rural areas for UN Africa Subregions: Population in millions and percentage in the urban, suburban, rural and hinterland classes in the five UN Africa Subregions.

UN Subregion	Population (millions) (population share)				
	Urban	Suburban	Rural	Hinterland	Total
Northern Africa	113.38 (46 %)	98.97 (40 %)	14.20 (6 %)	19.32 (8 %)	245.87
Western Africa	105.42 (26 %)	223.25 (56 %)	54.77 (14 %)	17.78 (4 %)	401.21
Middle Africa	40.72 (23 %)	72.38 (40 %)	35.09 (20 %)	31.18 (17 %)	179.37
Eastern Africa	53.26 (15 %)	201.73 (56 %)	66.65 (19 %)	38.44 (11 %)	360.08
Southern Africa	47.42 (31 %)	60.85 (40 %)	32.86 (22 %)	9.91 (7%)	151.04

To improve transparency, we compare our aggregated URCA classes and United Nations (UN) Population Division urbanization estimates in Table 2. This comparison shows that our "Suburban" class contains a larger proportion of the population compared to the UN "Towns" category (16% more on average), whereas our "Urban" class captures a smaller share relative to the UN "Cities" category (16% less on average). This suggests that the main difference arises from how peri-urban and connected settlements are classified, rather than from a systematic underestimation of rural populations.

Importantly, the purpose of this classification is not to reproduce official demographic statistics but to stratify populations by functional spatial accessibility to urban centers. The main conclusions remain robust under this interpretation: accessibility declines consistently along the continuum from urban and connected regions toward increasingly remote areas.

Table 2 Share of the population of each rural-urban class for the African UN subregions according to the United Nations' official estimates, and difference in share between the URCA 4-level division and the UN categories. The comparison uses the following mapping: cities = urban, towns = suburban, and rural UN = rural + hinterland. Positive differences indicate that the URCA class has a larger population share than the corresponding UN category, and negative values indicate a smaller share.

Subreg.	UN Division (%)			Difference (URCA – UN) (%)		
	Cities	Towns	Rural	Urban–Cities	Suburb.–Towns	Rural+Hint.–Rural UN
NA	57	29	14	-11	11	0
WA	43.6	29.3	27.1	-17.6	26.7	-9.1
MA	51.5	21.4	27.1	-28.5	18.6	9.9
EA	25.7	41.2	33.1	-10.7	14.8	-3.1
SA	46.5	29.2	24.4	-15.5	10.8	4.6
Average	–	–	–	-16.66	16.38	0.46

4 Summary of accessibility metrics

Table 3 Pros and Cons of market accessibility metrics: Schematic comparison of the advantages and disadvantages of the three accessibility metrics used in this work.

	**CUMULATIVE**	**TRAVEL TIME**	**ENTROPY**
Concept	Number of markets	Closeness	Spatial Distibution
Pros	1. Interpretable units<br>2. Considers quantity	1. Interpretable units<br>2. No parametric dependence	1. Smaller value range<br>2. Bridges cumulative and travel time
Cons	1. Bias towards populated areas<br>2. Arbitrary threshold radius	1. Does not consider quantity<br>2. Assumes consumers only travel to the closest market	1. Not interpretable units<br>2. Assumed travel behaviour in the absence of data<br>3. Lack of references in the literature

5 OSM and WFP data coverage in Ethiopia and Nigeria

To visualise complementarity between data sources, In Fig. 4 maps raw OpenStreetMap (OSM) amenities and WFP market points in Ethiopia and Nigeria before centroid clustering. WFP points concentrate in food-insecure or sparsely mapped regions (e.g., Somali Region in Ethiopia, north-east Nigeria), underscoring the value of merging the two datasets.

Figure 3 ranks all 54 African countries by the number of raw market points from each source (log-scaled axis). Disparities highlight where targeted data-improvement campaigns could most benefit accessibility estimates.

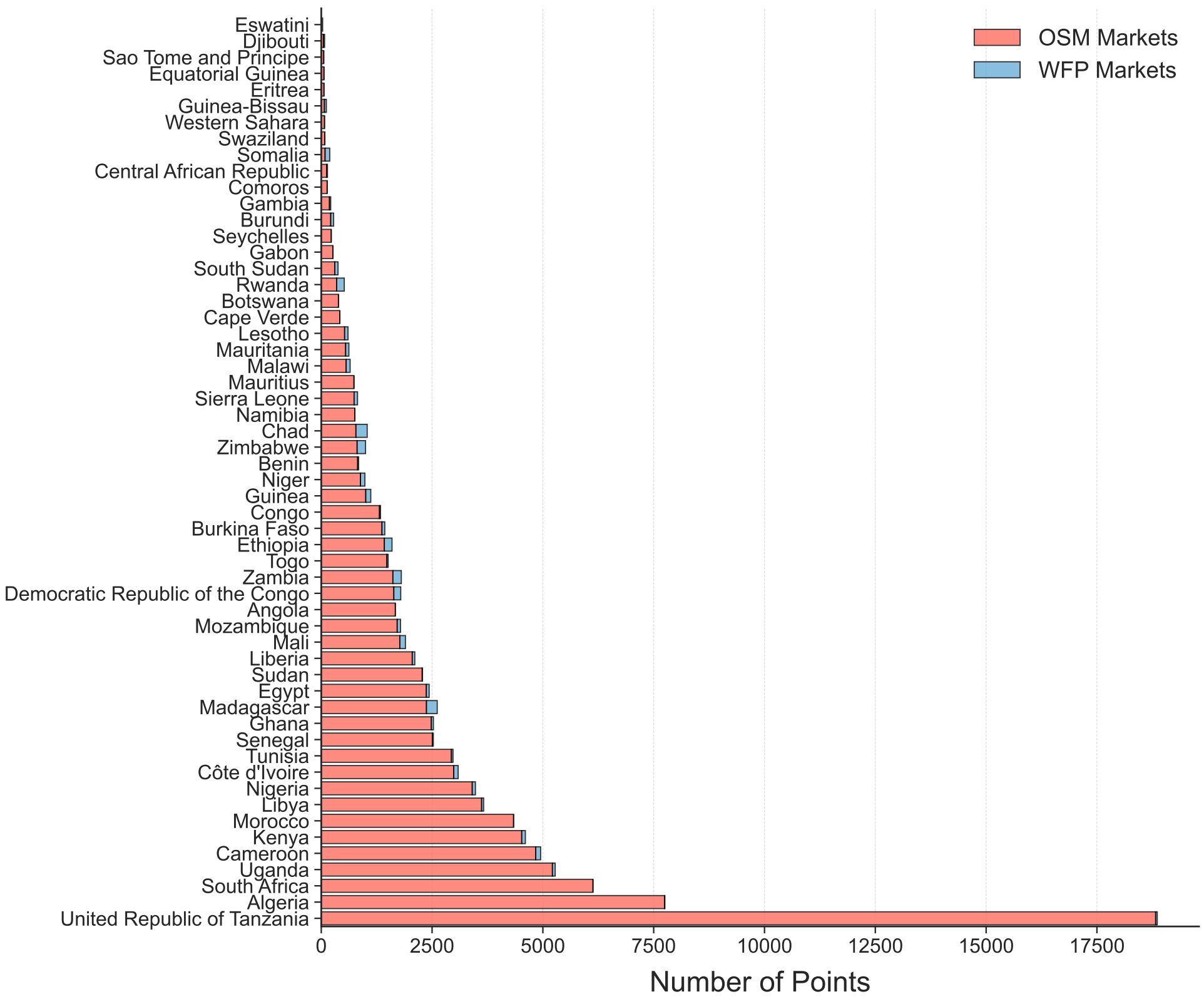

Fig. 3 Number of OSM (red) and WFP (blue) markets per country. We use a logarithmic y-axis for an improved visualization of the countries with WFP markets.

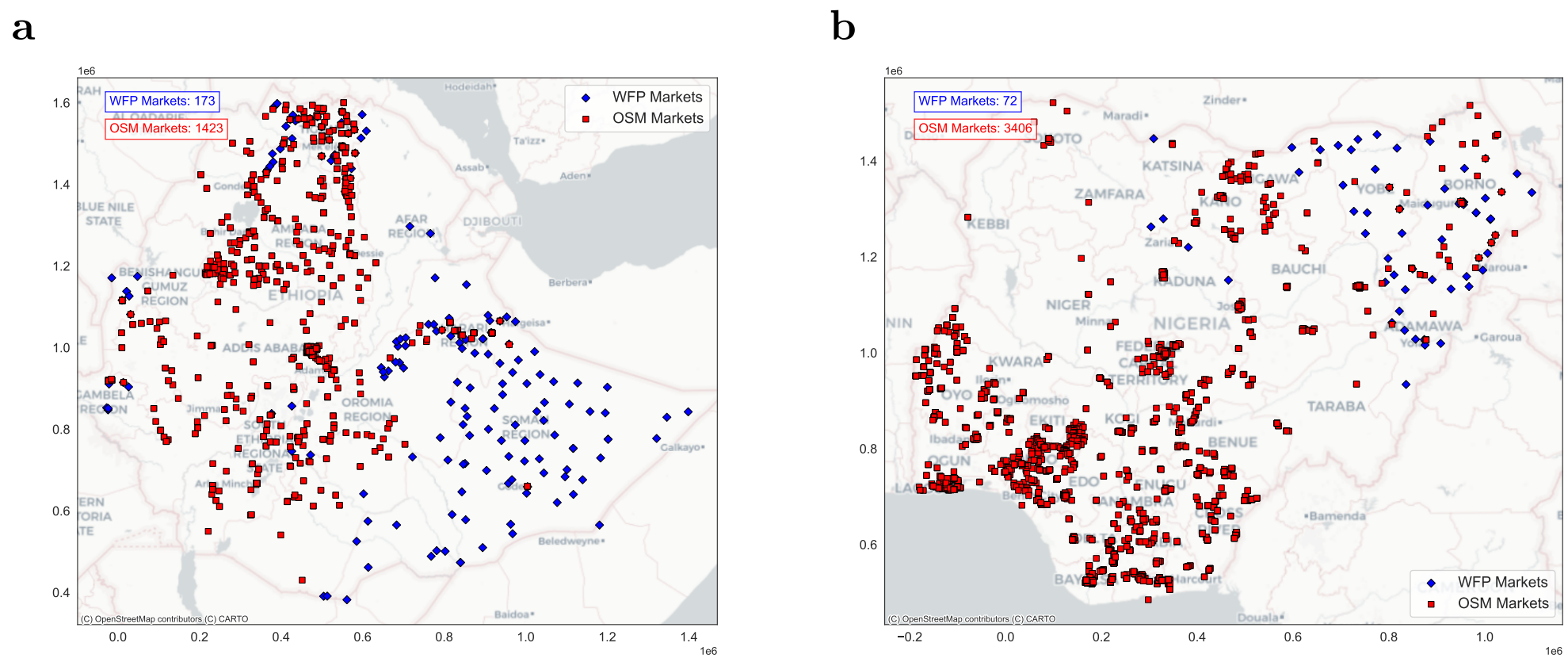

Fig. 4 Food shops and markets collected from OSM and WFP in Ethiopia (**a**) and Nigeria (**b**). These correspond to the raw data before the 15-minute clustering process.

6 Extracting market centroids

We transform raw points into market centroids by agglomerating amenities within a 15-min walking radius. Fig. 5 **a** shows the resulting centroids and Voronoi catchments for Addis Ababa. Figure 5 **b** plots the ratio nclusters/npoints versus candidate radii across Africa. Initially, for small cluster radius, the relative number of clusters drops significantly. The curve shows a slower decrease for 15 minutes and beyond, indicating that small changes in cluster radius do not significantly impact the resulting market distribution overall.

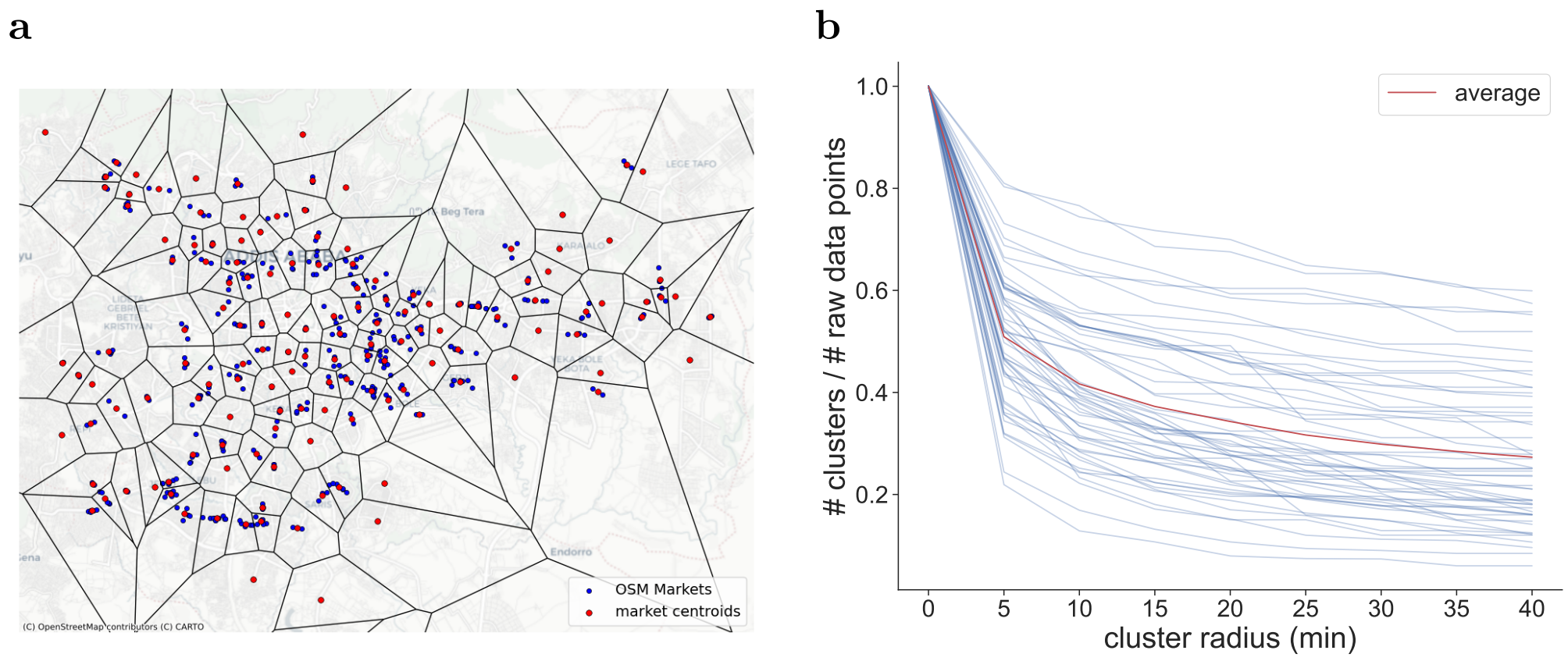

Fig. 5 **Clustering example and sensitivity analysis** (**a**) 15-minute cluster centroids of the OSM food shops with the respective Voronoi cells in Addis Abeba. Sensitivity analysis (**b**) of the number of clusters produced for different threshold radius for all African countries

7 Class distribution of IPC data

Figure 6 shows the class imbalance in IPC phases, with IPC phase 1 being the most prevalent across all Africa.

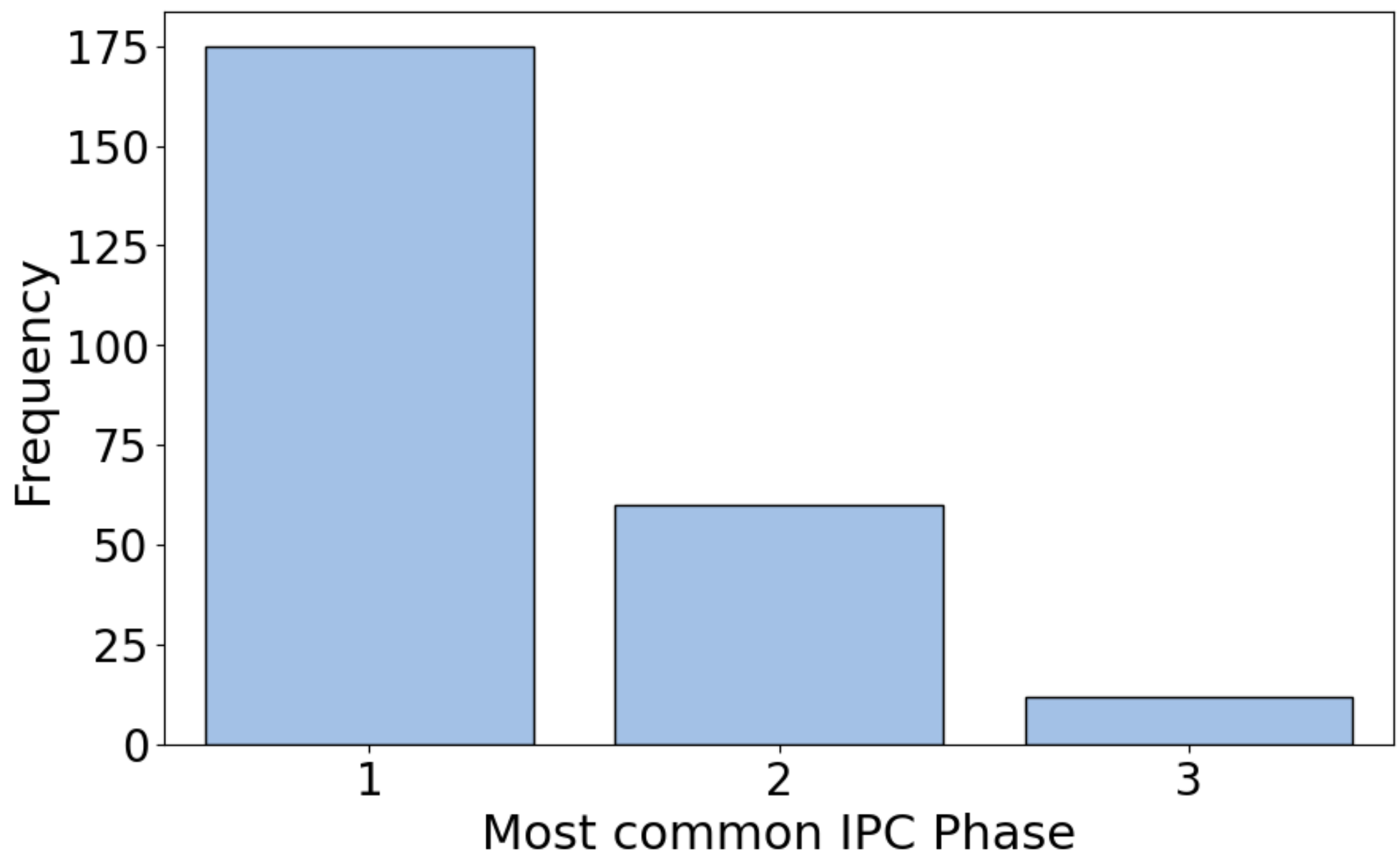

Fig. 6 Distribution of the most prevalent IPC phase by population across administrative level-1 regions in Africa, showing the number of regions in each phase.

8 External comparison of friction-surface travel-time estimates

To assess the robustness of the friction-surface-based travel-time estimates used in this study, we conducted an external comparison against travel times computed using a road-network-based routing engine. Specifically, we used the OpenRouteService (ORS) API, which calculates shortest-path travel times on the OpenStreetMap (OSM) road network.

8.1 Sampling procedure

We randomly sampled 100 origin–destination (OD) pairs across the study area. Origins correspond to randomly selected raster pixels from the 30 arc-second friction surface, while destinations correspond to randomly selected market locations derived from the harmonized OSM–WFP dataset. Because network-based routing requires mapped road connectivity, we restricted the sampled OD pairs to cases where both the origin and the destination were located within 2 km of a mapped road segment in OSM. This restriction ensures that ORS can compute a valid route. It should be noted that this constraint excludes some remote areas lacking mapped road infrastructure, where friction surfaces still allow travel-time estimation via terrain-based routing. For motorized travel, we computed travel times using ORS driving mode. For walking travel, we used the ORS walking profile. To facilitate interpretation, we report results both for the

full set of sampled OD pairs and for subsets restricted by Euclidean distance thresholds ($\leq$ 80 km for motorized travel; $\leq$ 20 km for walking), as extremely long-distance walking routes may produce unstable comparisons due to modeling differences.

8.2 Comparison methodology

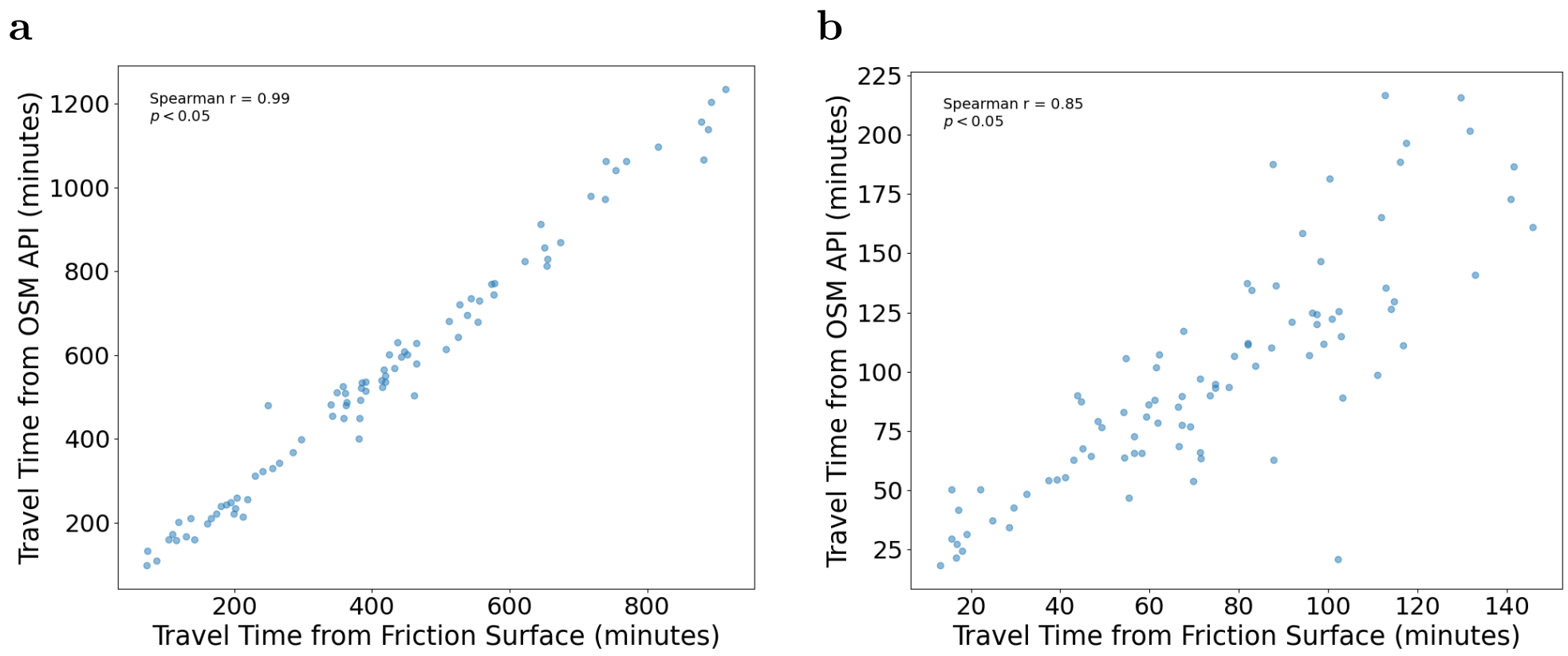

Fig. 7 Comparison between motorized travel times computed using the friction-surface approach (this study) and travel times obtained via OpenRouteService routing on the OpenStreetMap road network, based on 100 samples of origin–destination pairs. (**a**) Subset of OD pairs with Euclidean distance $\leq$ 80 km. (**b**) Full set of sampled OD pairs. Differences between estimates reflect the distinct modelling assumptions of continuous friction-surface routing versus road-network-constrained routing and potential incompleteness of the mapped road network.

For each OD pair, we computed:

- Travel time using the friction-surface approach described in the Methods (MCP_Geometric algorithm applied to motorized and walking friction rasters).
- Travel time using OpenRouteService routing on the OSM road network.

We then compared the two sets of estimates using scatterplots and correlation analysis. The results (Supplementary Figures X–Y) show a consistent positive association between friction-surface and network-based travel times across both motorized and walking modes. Differences between the two approaches are expected and reflect their distinct modeling assumptions:

1. Friction-surface routing represents a continuous cost landscape incorporating roads, land cover, slope, and off-road travel, allowing movement across all terrain types.
2. Network-based routing (ORS) is constrained to mapped road and pedestrian networks and may underestimate connectivity in areas where road mapping is incomplete.

Spearman correlation coefficients were $\rho = 0.99$ for unbounded pairs and $\rho = 0.85$ for a maximum OD separation of 80 km for motorized transport. For walking, $\rho = 0.85$ with a maximum separation between the origin and the market of 20 km. These results

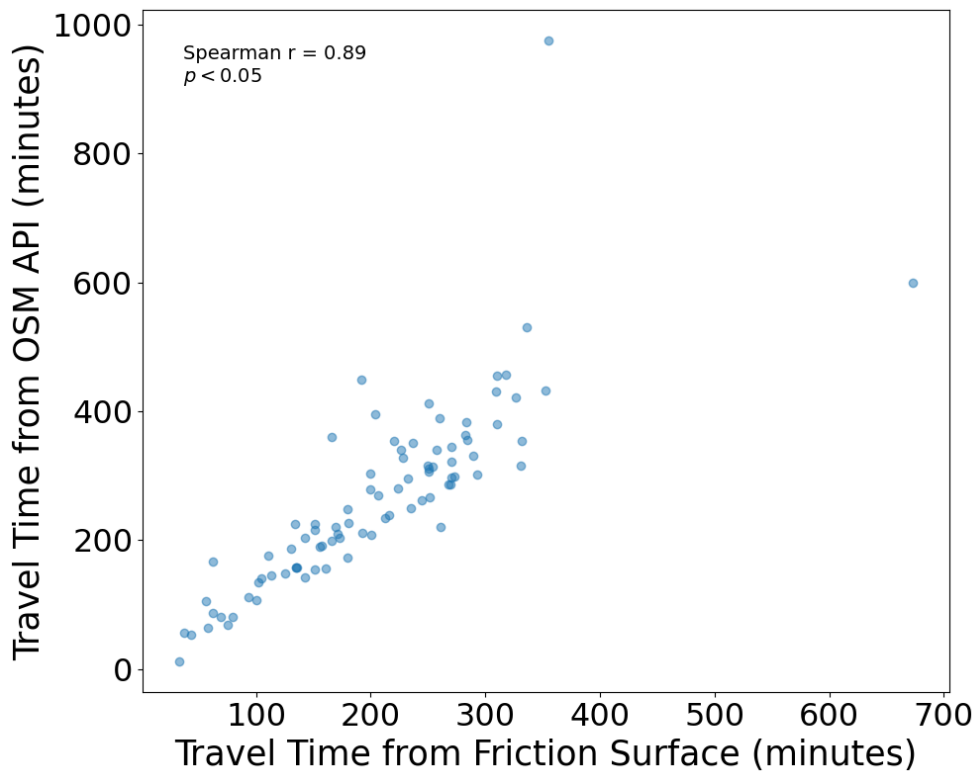

Fig. 8 Comparison between walking travel times computed using the friction-surface approach (this study) and travel times obtained via OpenRouteService routing on the OpenStreetMap pedestrian/road network, based on randomly sampled origin–destination pairs. Origins correspond to raster pixels and destinations correspond to randomly selected market locations. OD pairs were restricted to cases where both endpoints were within 2 km of a mapped road to ensure network routability. For interpretability, results are shown for OD pairs with Euclidean distance $\leq$ 20 km. Differences between estimates reflect the distinct modeling assumptions and potential incompleteness of mapped pedestrian infrastructure.

indicate substantial agreement between the two estimation approaches. Overall, the comparison indicates that while absolute travel-time estimates differ in some cases, the relative ranking of OD travel times remains broadly consistent across methods. This supports the robustness of the friction-surface approach for capturing large-scale spatial gradients in accessibility.
